# The ectopic expression of BRCA1 is associated with genesis, progression, and prognosis of breast cancer in young patients

**DOI:** 10.1186/1746-1596-7-181

**Published:** 2012-12-31

**Authors:** Qingli Zhang, Qinghui Zhang, Hua Cong, Xiaoli Zhang

**Affiliations:** 1 School of Medicine, Shandong University, Jinan, 250012, P.R. China

**Keywords:** BRCA1, WWOX, Gene detection

## Abstract

**Objective:**

The study is to explore the histopathological features and the molecular marker expression of young women with breast cancers.

**Methods:**

The pathological data of 367 cases of female breast cancer patients were retrospectively analyzed, focusing on the analysis of young breast cancer incidence trends and the clinical and pathological features.

**Results:**

Compared with elderly breast cancer patients, young women with breast cancers had larger tumor sizes, higher histological grades, and lymph node metastasis rates. The majority of patients were in the PTNM III stage, with the clinical and pathological features of strong invasiveness. The positive expression rate of the BRCA1 protein in the young group was higher than that in the old group. BRCA1 expression was positively correlated with the PTNM stage and axillary lymph node metastasis (*P* < 0.05).

**Conclusions:**

The ectopic expression of BRCA1 is associated with the genesis, progression, and prognosis of young breast cancer patients.

**Virtual slides:**

The virtual slide(s) for this article can be found here: http://www.diagnosticpathology.diagnomx.eu/vs/1628000054838044

## Introduction

Breast cancer has become one of the common diseases that seriously threaten women health. From 1982 to 2001, female breast cancer incidence showed an increasing trend in the Beijing urban area, with an average annual increase of 4.6% to 4.9% [1]. The age of onset of breast cancer in China is 8–10 years younger than in the Europe and the United States [2]. Women younger than 35 year old are less likely to get breast cancer according to clinical recordings. However, the incidence has an increasing trend with the increase of overall breast cancer patients in the recent years [2].

The breast cancer susceptibility gene (BRCA1), a tumor suppressor gene, encoded a factor inhibiting cell growth. The factor is also involved in cell cycle control, gene transcription regulation, DNA damage repair, apoptosis, and other important cellular processes. BRCA1 plays an important role in maintaining gene stability [3], with its mutation being related with 35%-40% of familial breast cancers and ovarian cancers. WWOX (WW domain containing oxidoreductase) is a new gene identified by Bednarek et al. using the shotgun gene sequencing technology combined with the separation and analysis of transcripts corresponding to the region of interest [4]. The WWOX gene is found with lost heterozygosity, abnormal transcription and low protein expression level in various kinds of tumors. The WWOX gene is considered to be a new tumor suppressor gene after discovery of the FHIT gene.

In this study, the expression of the breast cancer susceptibility gene BRCA1 and the tumor suppressor gene WWOX is detected using the immunohistochemical method in young and old female breast cancer patients. The mutation state of BRCA1 gene exon 2 and 20 was detected by PCR amplification and the direct sequencing testing method. The relationship between clinicopathological parameters and their clinical significance was analyzed.

## Materials and methods

### Materials

**Figure 1:**
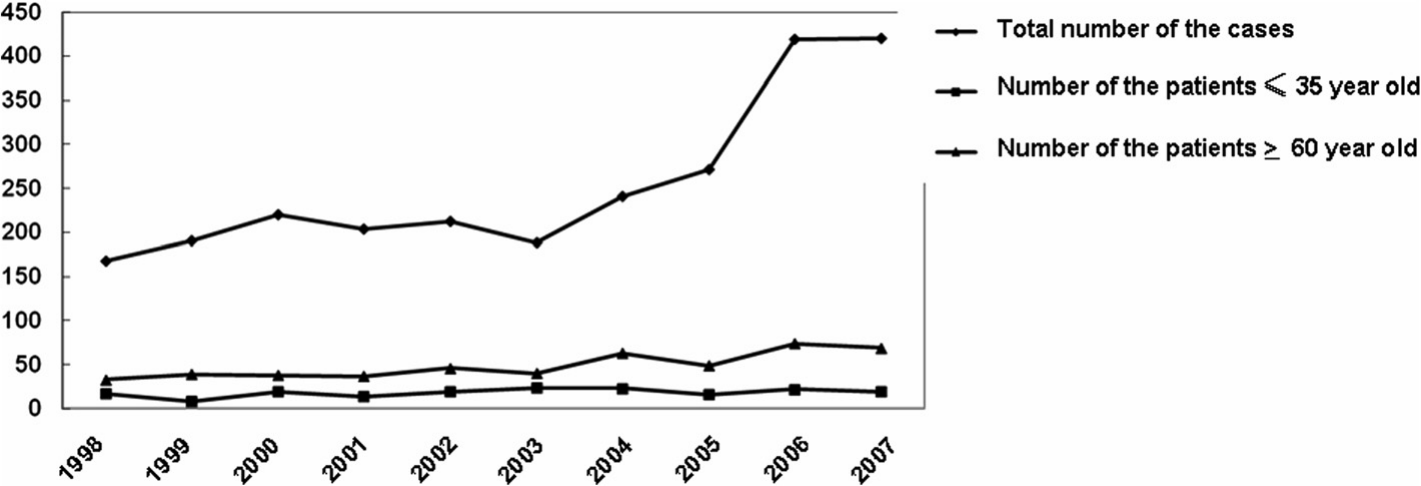
Analysis of numbers of the female breast cancer case, including the young and older breast cancer cases, diagnosed in the hospital in the past 10 years.

One hundred and eighty-one cases of patients (younger than 35 years), who undergone surgical resection or biopsy and confirmed by pathology to be breast cancer patients, were collected from January 1998 to December 2007 (Figure 1). They were from 19 to 35 years old, and the median age is 27 years old. One hundred and eighty-six cases of elderly female breast cancer patients (≥ 60 years) were randomly selected from the same period. They were from 60 to 85 years old, and the median age is 73 years old. The two groups of pathological data were analyzed retrospectively. Clinical and pathological characteristics of pathologic types, tumor sizes, histological grades, pathological stages, and lymph node metastasis were comparable. Pathological diagnosis and histological grades were determined using the World Health Organization breast tumor diagnostic criteria (2003). The UICC breast cancer pathological stage was determined using the pathological tumor-node-metastasis (PTNM) staging standard (2006). Prior written and informed consent was obtained from every patient and the study was approved by the ethics review board of Shandong University.

### Immunohistochemical method

The WWOX antibody was purchased from Beijing Boaosen Biotechnology Co., Ltd. BRCA1 antibody was purchased from Wuhan Boster Biological Engineering Co., Ltd. ER, PR, and Ki67 were expressed in the nucleus. HER2 was expressed in the cell membrane. BRCA1 was expressed in the nucleus or cytoplasm. BRCA1 staining criteria was described as follows: according to the expression of the area, < 5% of staining was recorded as (−); 5~20% of staining was recorded as (+); 21-50% of staining was recorded as (++); and > 50% of staining was recorded as (+++). WWOX was expressed in the cytoplasm. According to the expression extent, tumor cells without staining was recorded as (−); tumor cells with low staining was recorded as (+); medium staining was recorded as (+ +); and extensive staining was recorded as (+ + +).

### Genomic DNA Extraction and PCR Amplification

The Genomic DNA from breast cancer tissue was extracted using the TIANamp Genomic DNA extraction kit. The two pairs of sense and antisense oligonucleotide primers (Exon2F, GAAGTTGTCATTTTATAAACCTTT; Exon2R, TGTCTTTTCTTCCCTAGTATGT; Exon20F, ATATGACGTGTCTGCTCCACT; Exon20R, GGGAATCCA AATTACACAGC) used in PCR amplification were synthesized by Shanghai Biological Engineering Technology Co., Ltd (Shanghai, China).

### DNA sequencing

DNAStar MagAlign analysis software was used for sequence comparisons. Single nucleotide polymorphism (SNP) loci were searched. Data analysis of all the nucleic acid sequence was carried out according to the wild-type cDNA sequence of BRCA1 (U14680.1) in the GenBank. Translation from nucleotide to protein was compared using the Blast2 application program on the U.S. Center for Biotechnology Information (NCBI) website to determine whether the mutation sites will cause amino acid change.

### Statistical analysis

The application program SPSS16.0 was used for statistical analysis. Differences between the two groups were compared using the χ2 test. *P* < 0.05 was considered statistically significant. The correlation between two variables was determined using the Pearson parameter correlation analysis. *P* < 0.05 was considered for the two variables being related.

## Results

### General clinicopathological data

Among the patients being investigated, there were 160 single occurrence cases (88.4%) and 21 multiple occurrence cases (11.6%) in the young women group. There were 179 single occurrence cases and 7 multiple occurrence cases (11.6%) in the older women group. According to the pathological tumor-node-metastasis (PTNM) staging standard, tumor sizes were divided into the following grades: tumor size smaller than or equal to 2 cm (T1), tumor size larger than 2 cm, but smaller than or equal to 5 cm (T2), tumor size larger than 5cm (T3) (Table 1). Data comparison showed that more patients with T3 tumor size appeared in the young group than the older group. The difference was statistically significant (*P* < 0.05) (Table 1).

**Table 1:** Comparative analysis of the clinicopathological parameters in the young groups and old groups

**Parameters**	**Young (n %)**	**Old (n %)**	**x**^**2**^	***P***
Tumor size			7.053	0.029 *
≤2 cm	64 (35.4)	81 (43.5)		
>2 cm ≤5 cm	94 (51.9)	95 (51.1)		
>5 cm	23 (12.7)	10 (5.4)		
Pathological grading			21.354	0.0001*
I	4 (2.9)	19 (12.1)		
II	97 (69.3)	122 (77.7)		
III	39 (27.9)	16 (10.2)		
PTNM	18.290	0.0001*		
0~I	32 (17.7)	59 (31.7)		
II	89 (49.2)	97 (52.2)		
III	60 (33.1)	30 (16.1)		
Axillary lymph node metastasis			18.707	0.0001*
yes	106 (58.6)	67 (36.0)		
No	75 (41.4)	119 (64.0)		
Axillary lymph node			20.801	0.0001*
Transferring positive number				
0	75 (41.4)	119 (64.0)		
1~3	47 (26.0)	37 (19.9)		
4~9	28 (15.5)	16 (8.6)		
≥10	31 (17.1)	14 (7.5)		

Note: *, *P* < 0.05.

The number of breast cancer cases, diagnosed in our hospital, has significantly increased year by year between 1998 and 2007, with no significant changes in the case numbers of the young patient group, but slight increases in the case numbers of the older patient group (Figure 1). In both of the two groups, invasive ductal carcinoma is the most common histopathologic type, followed by ductal carcinoma in situ and invasive lobular carcinoma. Invasive ductal carcinoma was graded according to the amount of duct formation in the tumor tissues, the sizes of tumor cell atypia and the numbers of mitotic nucleus by reference to the Elston and Eliis histological grading criteria. It was found that patients in the younger group have more grade III tumors than the older group. The difference was statistically significant (*P* < 0.01) (Table 1).

### Immunohistochemical results

**Figure 2:**
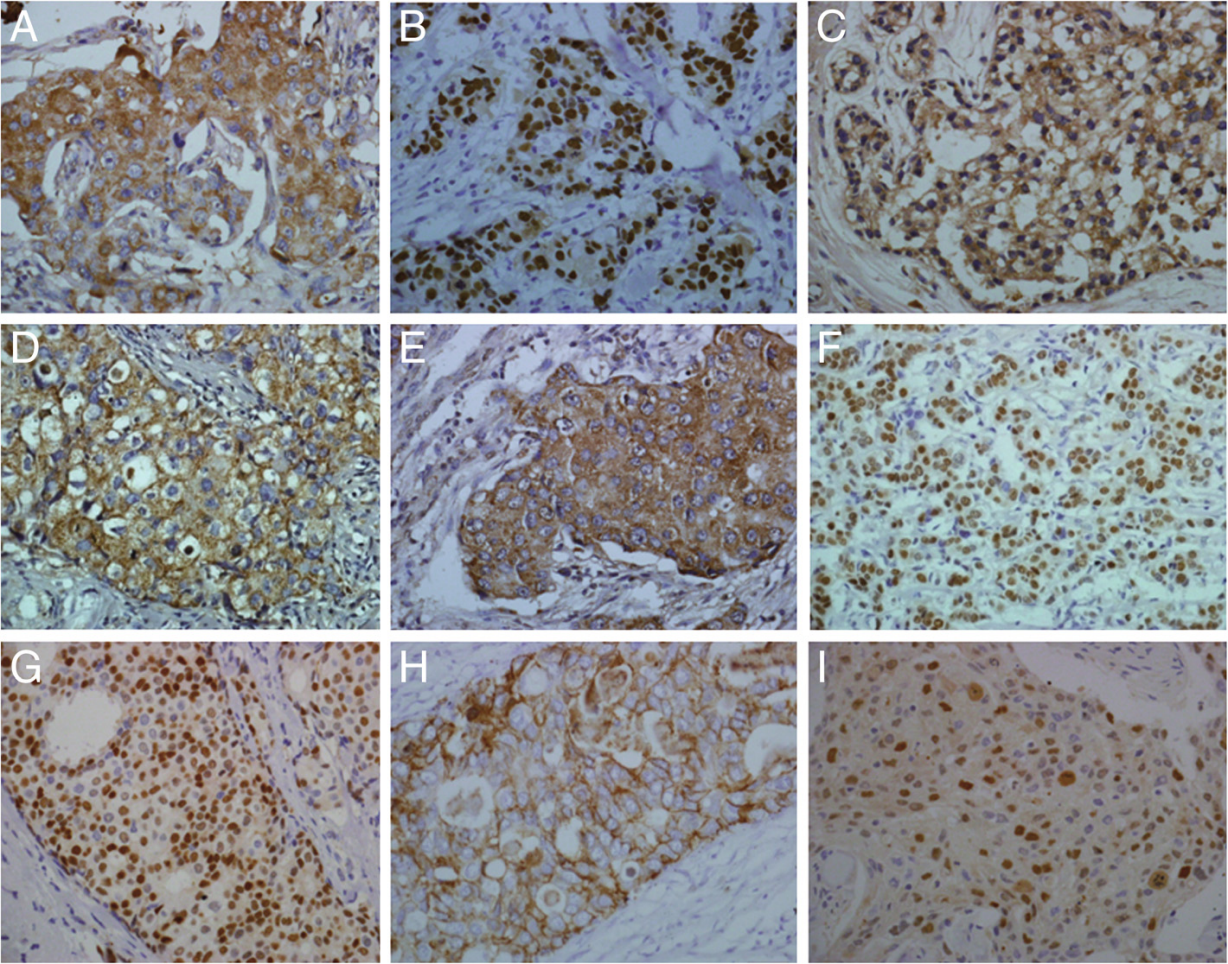
**A (young group) BRCA1 protein immunohistochemical staining was positive.** The positive products localized in the cytoplasm (× 400). **B** (young group) BRCA1 protein immunohistochemical staining was positive. The positive products localized in the nucleus (× 400). **C** (old group) BRCA1 protein immunohistochemical staining was positive. The positive products localized in the cytoplasm (× 400). **D** (young group) WWOX protein immunohistochemical staining was positive. The positive products localized in the cytoplasm (× 400). **E** (old group) WWOX protein immunohistochemical staining was positive. The positive products localized in the cytoplasm (× 400)**. F** (young group) ER protein immunohistochemical staining was positive. The positive product positioning in the nucleus (× 400). **G** (young group) positive immunohistochemical staining of PR proteins. The positive products localized in the nucleus (× 400) **H** (young group) C-erbB2 protein immunohistochemical staining was positive. The positive products localized in the membrane (× 400). **I** (young group) Ki67 protein immunohistochemical staining was positive. The positive products localized in the nucleus (× 400).

The positive protein expression rates of ER, PR, C-erbB2, BRCA1, and WWOX and the high proliferative rate of Ki67 in the young group were as follows: 66.4% (71/107), 54.2% (58/107), 27.1% (29/107), 65.4% (70/107), 86.9% (93/107) and 59.8% (64/107). The positive protein expression rates of ER, PR, C-erbB2, BRCA1 and WWOX and the high proliferative rate of Ki67 in the older group were as follows: 72.3% (81/112), 40.2% (45/112), 17.0% (19/112), 35.7% (40/112), 86.6% (97/112), and 55.4% (62/112). The Χ2 test results showed that the BRCA1 protein expression has statistically significant differences between the two groups (*P* < 0.01). The positive expression rate of BRCA1 protein in the younger group was higher than that in the older group (Table 2). Both of the expression sites of the two groups are in the cytoplasm. ER, PR, HER2, and WWOX protein expression and Ki67 high proliferation rate had no significant differences between the two groups (*P* > 0.05). The immunohistochemical pictures of ER and PR, HER2, Ki67, BRCA1 and WWOX protein expression of the young group and the old group are shown in Figure 2.

**Table 2:** Comparative analysis of the immunohistochemical results of young and old patient groups

**Indicators**	**Young group (%)**	**Old group (%)**	**X**^**2**^	***P *value**
BRCA1			53.206	0.001*
—	37 (34.6)	72 (64.3)		
+	13 (12.1)	30 (26.8)		
++	38 (35.5)	10 (8.9)		
+++	19 (17.8)	0 (0)		
WWOX			4.610	0.203
—	14 (13.1)	15 (13.4)		
+	56 (52.3)	56 (50.0)		
++	32 (29.9)	27 (24.1)		
+++	5 (4.7)	14 (12.5)		
ER			3.867	0.276
—	36 (33.6)	31 (27.7)		
+	48 (44.9)	48 (42.9)		
++	16 (15.0)	28 (25.0)		
+++	7 (6.5)	5 (4.5)		
PR			5.215	0.157
—	49 (45.8)	67 (59.8)		
+	48 (44.9)	34 (30.4)		
++	7 (6.5)	7 (6.2)		
+++	3 (2.8)	4 (3.6)		
C-erbB2			4.012	0.235
—	63 (58.9)	81 (72.3)		
+	15 (14.0)	12 (10.7)		
++	18 (16.8)	9 (8.1)		
+++	11 (10.3)	10 (8.9)		
Ki67			2.128	0.546
<15%	43 (40.2)	50 (44.6)		
≥15%	64 (59.8)	52 (55.4)		

### Correlation analysis of patient’s parameters

BRCA1 expression was positively correlated with PTNM stages (r = 0.158, *P* < 0.05), positively correlated to Axillary lymph node metastasis (r = 0.202, *P* < 0.01), (Table 3) and has no correlation with ER, PR, HER2, and WWOX expression, Ki67 proliferation rate and the histological grade (*P* > 0.05). The WWOX expression has no correlation with various clinical indicators (P > 0.05) (Table 4).

**Table 3:** Analysis of the correlation between the BRCA1 results and clinicopathological parameters in the young groups and old groups

**Items**	**Young groups (n) BRCA1**		**Old groups (n) BRCA1**		**r**	**P**
	—	+	—	+		
ER					0.042	0.540
—	13	23	21	10		
+	24	47	51	30		
PR					0.073	0.282
—	20	29	42	25		
+	17	41	30	15		
C-erbB2					0.051	0.452
—	29	49	59	34		
+	8	21	13	6		
Ki67					0.074	0.272
<15%	12	31	29	21		
≥15%	25	39	43	19		
WWOX					−0.145	0.128
—	6	8	7	8		
+	31	62	65	32		
Histological grades					0.077	0.260
I	2	1	10	9		
II	26	43	53	28		
III	9	26	9	3		
PTNM					0.158	0.020 *
0~I	5	9	25	11		
II	21	34	38	21		
III	11	27	9	8		
Axillary lymph node metastasis					0.205	0.002*
Yes	23	48	22	18		
No	14	22	50	22		

Note: *, P < 0.05.

**Table 4:** Analysis of the correlation between the WWOX results and clinicopathological parameters in the young groups and old groups

**Items**	**Young groups(n) WWOX**		**Old groups(n) WWOX**		**r**	**P**
	—	+	—	+		
ER					0.118	0.080
—	2	34	5	26		
+	12	59	10	71		
PR					0.113	0.094
—	6	43	11	56		
+	8	50	4	41		
C-erbB2					0.033	0.629
—	11	67	14	79		
+	3	26	1	18		
Ki67					0.010	0.987
<15%	6	37	11	4		
≥15%	8	56	39	58		
Histological grades					0.016	0.819
I	1	2	4	15		
II	11	58	11	70		
III	2	33	0	12		
PTNM					−0.085	0.207
0~I	1	13	3	33		
II	8	47	10	49		
III	5	33	2	15		
Axillary lymph node metastasis					0.099	0.144
Yes	12	59	8	32		
No	2	34	7	65		

**Figure 3:**
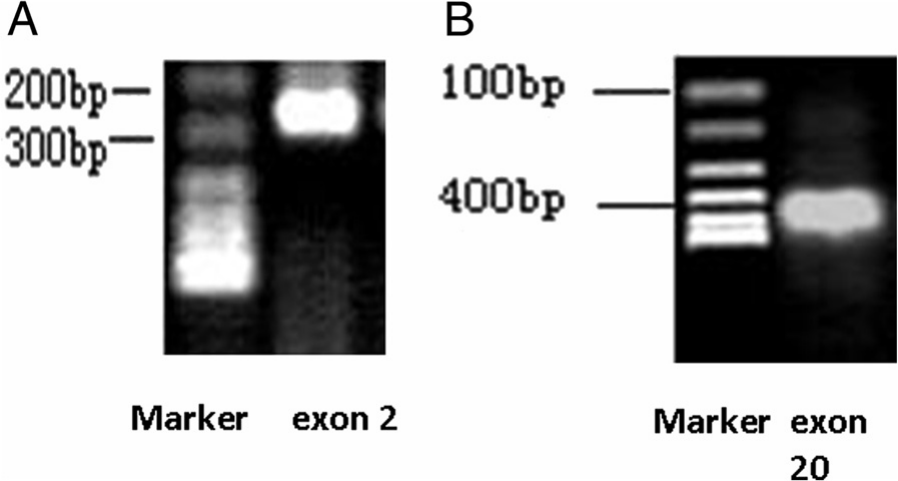
**A The PCR product of exon 2 of the BRCA1 gene (259 bp). B** The PCR product of exon 20 of the BRCA1 gene (401 bp).

To amplify the BRCA1 gene 2 and 20 exon, we performed PCR using two pairs of sense and antisense oligonucleotide nucleotide primers. The amplification results showed that both of them showed single bands (Figure 3).

### DNA sequencing results

**Figure 4:**
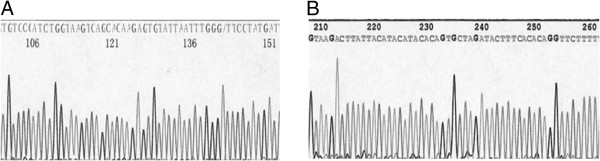
**A The partial DNA sequencing results of exon 2 of BRCA1 gene of patient 6. B** The partial DNA sequencing results of exon 20 of BRCA1 gene of patient 6.

The BRCA1 gene has 22 coding exons. We carried out DNA sequencing around the gene mutation hot spots: exon 2 and exon 20 in BRCA 1 gene. The exon 2 is 259 bp and the exon 20 is 401 bp, which account for 11.8% of the total sequence length in exon (660/5600). Sequence comparison by the DNAStar and single nucleotide polymorphism (SNP) sites searching method showed no sequence variation. The partial DNA sequencing results of exon 2 and exon 20 of BRCA1 gene of patient 6 was shown in Figure 4.

## Discussion

Suris-Swarm [5] reported that women younger than 50-year-old suffering from 4.3 times higher the possibility of getting bilateral primary breast cancer (BPBC) than women over the age of 50 year old. Award reported that women less than 40-year-old are in the population having a high risk of getting BPBC [6]. Among the patients studied in this paper, the diseased tissues are more frequently found in left breasts than the right breasts of patients in the younger group. Two (1.9%) patients get tumors on both of the left and right breasts in the young group, while no patient get tumors on both of the left and right breasts in the older group. The majority of patients have their incidence positions at the outer upper quadrant, followed by the inner upper quadrant and the nipple. Triple-negative breast cancer (TNBC) represents the major phenotype of basal-like molecular subtype of breast cancer, characterized by higher incidence in young women and a very poor prognosis. Svoboda [7] found that expression of miR-34b negatively correlates with an overall survival of TNBC patients.

Hutter [8] found that the number of lymph node metastasis is one of the decisive factors affecting the prognosis of breast cancer. Patients without lymph node metastasis have more favorable prognosis than those having lymph node metastasis. The more the number of metastatic lymph nodes, the worse the prognosis. Xu [9] found that claudin-6 is an important factor influencing lymphatic metastasis, whereas up-regulation of HDAC1 is associated with tumor progression and invasiveness in breast IDC. According to Liu et al. [10], the 3-year -and 5-year survival rates of young breast cancer patients with lymph node metastasis was 61.11%, 25% and the 3-year and 5-year survival rates of young breast cancer patients without lymph node metastasis was 100% and 83.33%, respectively. It is reported that young breast cancer patients have higher lymph node metastasis rates. More lesions are located in the internal mammary areas and the patients have late clinical stage, higher rate of infiltrating tumor occurrence, strong tumor invasiveness, easy transfer, and poor prognosis [11,12]. According to our research, the younger group has more cases with tumor diameter greater than 5 cm than the older group (*P* < 0.05). The young group has more histological grade III tumors than the older group (*P* < 0.01),which is consistent with finding reported by Jimor [13] and Chen [14]. Since the pathological stage is a judgment made after visual inspection of the postoperative pathological specimens, and can more accurately reflect the severity and extent of breast cancer, it is more accurate than clinical staging.

In this study, according to the PTNM staging, the young group has less patients in phases 0 - I and phase II than the older group, and the numbers of patients in phase III was significantly more than the elderly group. Two groups have a significant statistical difference (*P* < 0.01). The younger group has higher axillary lymph node metastasis rates and the proportion of positive lymph node was significantly higher than the older group (*P* < 0.01), which is consistent with previous reports. In this study, we found young women with breast cancer have larger tumor mass, higher histological grade and lymph node metastasis rate than the elderly group. The majority of patients fall into the PTNM stage III.

As a tumor suppressor gene, the breast cancer susceptibility gene l (BRCA 1), not only inhibits cell growth, but is also involved in cell cycle regulation, gene transcription, DNA damage repair and apoptosis, and some other important cellular activities. It plays an important role in maintaining genetic stability. There is study found that the BRCA1 protein exits in the nucleus of normal mammary epithelial cell and the cytoplasm of tumor cells [15]. Chen et al. [16] find that dislocation of the cytoplasmic BRCA1 protein in breast cancer cells is related to the occurrence and metastasis of breast cancer, but the molecular mechanism is unclear. Fu [17] and Liu [18] found the BRCA1 protein is highly expressed in breast cancer, suggesting that the abnormal expression of the BRCA1 has some correlations with the occurrence and growth of breast cancer. Liu [18] also found BRCA1 protein expression and the patient age were negatively correlated. Younger patients have higher BRCA1 protein expression rate. In our study, BRCA1 protein is expressed in the cytoplasm of breast cancer cells of both of the young group and elderly group. The young group has higher positive expression rate than the old age group (< 0.01). BRCA1 protein expression is positively correlated with PTNM stage and axillary lymph node metastasis, which is consistent with the results reported by Chen [16] and Liu [18]. The younger group has a higher degree of malignancy and poor prognosis. The ectopic expression of BRCA1 in breast cancer is closely related to the happening, development and prognosis of young breast cancer patients.

Tomasz [19] found that methylation of the BRCA1 gene in PB DNA correlates with increased risk of breast cancer, suggesting that aberrant methylation of genes in PB and disease predisposition are related. Lv [20] observed that EGFR gene mutations were rare in breast carcinomas, but EGFR gene amplification was detected in about one third of the cases in this population. In their study, rare mutations in the EGFR gene in patients with breast cancer were detected, indicating that EGFR gene mutations are infrequent in this cohort of breast cancers. We carried out DNA sequencing on the gene mutation hot spots, the exon 2 and exon 20, in BRCA 1 gene. Fresh specimens of 10 cases of unrelated young invasive ductal carcinoma was compared by DNA sequencing to search for single nucleotide polymorphism (SNP) sites, the results found no sequence variation.

WWOX protein contains 414 amino acids. In its amino-terminal, there are two WW domains. The WW functional domains are related to the interaction between the proteins [21]. Protein interactions are necessary for the tumor suppressor genes to inhibit tumor growth through signal transduction pathways. The deletion and mutation of WWOX may inhibit apoptosis and promote tumor occurrence and development [22]. In this study, WWOX protein expression of the young group and old group were 86.9% and 86.6%, respectively. The difference was not statistically significant (*P* > 0.05), the experiments also showed no correlation (P > 0.05) between WWOX and age and other clinical indicators.

Patient age itself is not a major factor affecting the prognosis of breast cancer. In young patients with poor prognosis, it is mainly due to their adverse pathological parameters and invasive biological characteristics. Therefore, clinically, comprehensive analysis should be carried on age and tumor clinicopathological and biological indicators.

## Conclusions

The ectopic expression of BRCA1 is associated with the genesis, progression, and prognosis of young breast cancer patients.

## Competing interests

The authors declare that they have no competing interests.

## Authors’ contributions

All authors read and approved the final manuscript.
